# Inhibitory effect and mechanism of action of Carvacrol as a promising natural food preservative against *Fusarium acuminatum* causing postharvest rot of garlic scapes (*Allium sativum* L)

**DOI:** 10.3389/fmicb.2025.1637313

**Published:** 2025-09-10

**Authors:** Pei Li, Jing Liu, Liqun Zhang, Wenqing Wu, Can He, Boxi Tan, Shijing Tang, Lu Yu

**Affiliations:** ^1^Guizhou Key Laboratory of Miao Medicine, Qiandongnan Engineering and Technology Research Center for Comprehensive Utilization of National Medicine, Kaili University, Kaili, China; ^2^School of Liquor and Food Engineering, Guizhou University, Guiyang, China; ^3^Guizhou Southern Dairy Co., Ltd., Guiyang, China

**Keywords:** inhibitory effect, mechanism of action, carvacrol, *Fusarium acuminatum*, garlic scapes

## Abstract

During prolonged storage of garlic scapes (*Allium sativum* L.), the proliferation of microorganisms, particularly fungi, frequently causes postharvest rot, leading to moss-covered stem spots, tissue softening, depression, and even structural breakage. Carvacrol, a promising natural food preservative, exhibits various biological activities against different microorganisms. To investigate the inhibitory effects and mechanism of action of carvacrol against specific pathogens responsible for postharvest rot in garlic scapes, in this study, a specific pathogenic fungal strain responsible for postharvest rot in garlic scapes, designated as strain *F*, was initially isolated from symptomatic garlic scapes and identified as *Fusarium acuminatum* through a combination of morphological, physiological, and molecular biological analyses. Meanwhile, our findings revealed that carvacrol can significantly delay the onset of postharvest rot symptoms in garlic scapes and exhibit potent in vito inhibitory activity against *Fusarium acuminatum*, with a median effective concentration (EC_50_) of 36.17 μg/L. In addition, scanning electron microscope (SEM) observations indicated that carvacrol could induce irreversible alterations in the morphology and structure of the hyphae, leading to deformation and rupture. Furthermore, the combined transcriptome and proteome analysis results indicated that carvacrol primarily affects the steroid biosynthesis and MAPK signaling pathway cell signaling pathways in *Fusarium acuminatum* to interference compromises the integrity and stability of the cell membrane, consequently suppressing the growth and proliferation of *Fusarium acuminatum*.

## 1 Introduction

Fungi can contaminate a wide range of agricultural commodities both pre- and post-harvest, making them the most prevalent food spoilers (Pouris et al., 2024; Bento de Carvalho et al., 2024). Notably, certain species, such as *Fusarium* sp., *Penicillium* sp., and *Aspergillus* sp., are capable of producing mycotoxins (Rahman et al., 2020). Due to their diverse toxic effects and high thermal stability, these mycotoxins pose significant health risks to both humans and animals (Janik et al., 2020). Factors contributing to fungal growth and mycotoxin production include poor harvesting practices, inadequate storage, suboptimal transportation, marketing, and processing conditions (Wagacha and Muthomi, 2008; Osei-Kwarteng et al., 2024). Despite advancements in food production techniques, food safety remains a critical public health concern (King et al., 2017; Li S. B. et al., 2021). It is estimated that up to 30% of individuals in industrialized countries experience a foodborne illness annually.

Garlic scapes (*Allium sativum* L.), the flower stalks from the seed heads of garlic bulbs, are native to West Asia or Europe and have gained global cultivation (Kovarovič et al., 2019; Shemesh-Mayer et al., 2023). The garlic scapes are distinguished by their crisp texture along with tender juiciness while being rich in cellulose, vitamin C (Vc), allicin, polysaccharides, minerals, and other vital nutrients (Li G. Q. et al., 2021). The consumption of garlic scapes has recently increased as it has gained popularity because of its excellent nutrient profile (Borlinghaus et al., 2014; Deng et al., 2023). As more consumers demand convenient, fresh, and healthy foods, fresh-cut garlic scapes can be expected to become a profitable agricultural commodity. However, during extended storage periods of garlic scapes, the proliferation of microorganisms, especially fungi, often leads to postharvest rot, resulting in stem spots covered with mosses, tissue soft rot, depression and even breakage (Chen et al., 2019).

The use of natural products from inherently disease-resistant plants to combat pre- and post-harvest diseases represents an innovative strategy in sustainable agricultural development. This approach is safer than conventional chemical products because it exhibits lower toxicity to natural enemies, humans, and other mammals. Carvacrol (5-isopropyl-2-methylphenol, C_10_H_14_O), a phenolic monoterpene compound with a free hydroxyl group, is naturally found in the essential oils of oregano (*Origanum vulgare*), thyme (*Thymus vulgaris*), pepperwort (*Lepidium flavum*), wild bergamot (*Citrus aurantium*), and other plants (Mączka et al., 2023). Carvacrol has been produced by chemical and biotechnological synthesis via metabolic engineered microorganisms (More et al., 2007). Previous studies have shown that carvacrol exhibits diverse biological activities, such as antifungal, antibacterial, antioxidant, and anticancer properties (Veldhuizen et al., 2006; Mączka et al., 2023). Due to its flavoring (oregano-like smell and pizza-like flavor) and antifungal properties, it is most often used in in controlling fungal decay in postharvest agricultural products as a natural food preservative (Abbaszadeh et al., 2014). Moreover, it has been classified as generally recognized as safe by the U.S. Food and Drug Administration (FDA), and it is currently employed in the food industry as a Category B chemical flavoring agent that may be added to foodstuffs at a level of 2 ppm in beverages, 5 ppm in flakes, and 25 ppm in candies (Yang et al., 2024; Addo et al., 2023; Imran et al., 2022).

In this study, the preservative effect analysis of carvacrol against postharvest rot of garlic scapes was performed. Meanwhile, a specific pathogenic fungal strain was isolated from symptomatic garlic scapes from Guizhou Province, China and identified using a combination of conventional identification method and molecular analysis technique. Additionally, the inhibitory effect and mechanism of action of carvacrol against the specific pathogenic fungal strain was investigated utilizing the combined transcriptome and proteome analysis.

## 2 Materials and methods

### 2.1 Sample collection

To evaluate the preservative effects of carvacrol and identify the specific pathogens responsible for postharvest rot in garlic scapes in China, a total of approximately 300 asymptomatic and symptomatic garlic scapes (“Chaohua” cultivar), produced in Guizhou Province, were collected from various vegetable markets in July 2023.

### 2.2 Determination of preservative effect

#### 2.2.1 Determination of decay rate

The asymptomatic garlic scapes were surface-sterilized with 75% ethanol followed by sterilized distilled water, and subsequently air-dried on a clean bench. The sterilized garlic scapes were then sprayed with carvacrol at concentration of 100 μg/L, and air-dried again on a clean bench before being incubated at 28 °C with 95% relative humidity. The decay rate analysis was performed in triplicates with 30 randomly sampled for each replicate. The decay rates were observed and recorded at 5, 10, 15, and 20 days post-treatment using the following formula (Liu et al., 2022).


$$\text{Decay rate}\;(\%)=\frac{\text{Number of rotten garlic scapes}\;}{\text{Number of garlic scapes}}\times 100\%$$

#### 2.2.2 Determination of weight loss

The weight losses of the postharvest garlic scapes after 5, 10, 15, and 20 days post-treatment were determined using a digital balance and expressed in percentage using the following formula (Owolabi et al., 2021).


$$\text{Weight loss}\;(\%)=\frac{\text{Initial weight}-\text{Weight}\;\mathrm{at}\;\text{sample time}\;}{\text{Initial weight}}\times 100\%$$

#### 2.2.3 Determination of vitamin C (Vc) and soluble protein (SP) contents

The Vc contents in postharvest garlic scapes were determined using the molybdenum blue spectrophotometry (He et al., 2021). Fresh garlic scape samples (0.5 g), collected at 5, 10, 15, and 20 days post-treatment, were homogenized with 25 mL of oxalic acid-EDTA solution (w/v). The homogenate was filtered, and 10 mL of the filtrate was mixed with 1 mL of phosphate-acetic acid buffer, 2 mL of 5% sulfuric acid, and 4 mL of ammonium molybdate solution. The Vc contents were then measured using an UV-6000 ultraviolet–visible (UV)-spectrophotometer (Shimadzu, Japan) at a wavelength of 705 nm.

The SP contents in postharvest garlic scapes were determined using the Coomassie brilliant blue G-250 dye-binding method (Murphey et al., 1989). Fresh garlic scape samples (0.5 g), collected at 5, 10, 15, and 20 days post-treatment, were homogenized in 8 mL of distilled water. The homogenate was centrifuged at 3000×*g* for 10 min at 4 °C. Subsequently, 0.2 mL of the supernatant was mixed with 0.8 mL distilled water and 5 mL of Coomassie brilliant blue G-250 solution. The absorbances were measured at 595 nm using an UV-spectrophotometer (UV-6000, Shimadzu, Japan) after a 5 min incubation period.

#### 2.2.4 Determination of polyphenol oxidase (PPO) and malonaldehyde (MDA) content

The contents of PPO and MDA of the postharvest garlic scapes were detected using the commercially available enzyme assay reagent kits produced by Suzhou Geruisi Biotechnology Co., Ltd. (Suzhou, China) (Fan et al., 2022).

### 2.3 Pathogen isolation and molecular characterization

#### 2.3.1 Pathogen isolation

Small sections of the infected base, stem, and apical regions of symptomatic garlic scapes were surface sterilized using 75% (v/v) ethanol and subsequently rinsed three times with sterile distilled water. The sterilized tissue samples were then placed on potato dextrose agar (PDA, 6 g potato powder, 20 g glucose, 20 g agar, 1 L sterile distilled water) plates and incubated at 28 °C for 72 h. Hyphae emerging from the tissue samples were aseptically transferred using an inoculation loop to fresh PDA plates and incubated at 30 °C for 48–72 h. Individual hyphal colonies were selected and sub-cultured on fresh PDA plates twice to ensure purity, and the purified cultures were stored at 4 °C for subsequent use.

#### 2.3.2 Pathogenicity test

The pathogenicity tests of the isolated specific pathogen were conducted by inoculating a conidial suspension (1.0 × 10^6^ conidia/L) onto the basal, stem, and apical regions of 20 fresh garlic scapes (“Chaohua” cultivar). After an incubation period of 7 days in an incubator set at 28 °C with 95% relative humidity, rot symptoms infected by the isolated pathogen resembling those observed in the collected samples were observed in the base, stem, and apical of garlic scapes. The isolated pathogen re-isolated from the symptomatic garlic scapes based on Koch’s postulates was subsequently selected for further characterization through morphological characterization and sequencing.

#### 2.3.3 Morphological characterization

After 72 h of growth on the PDA plate, the morphological characterization of the specific pathogen was observed with the naked eye and under an optical microscope.

#### 2.3.4 Molecular biological characterization

Approximately 25 mg of the specific pathogen were collected for genomic deoxyribonucleic acid (DNA) extraction using TIANamp fungal DNA distilling kit (Tiangen-Biotech Corporation Ltd., Beijing, China) and DNA concentration and quality were estimated using an ASP-3700 spectrophotometer (ACTGene, Piscataway, NJ, USA). Molecular identification was confirmed by sequencing the rDNA internal transcribed spacer (ITS) using primers ITS1/ITS4 (ITS1: 5′-TCCGTAGGTGAACCTGCGG-3′, ITS4: 5′-TCCTCCGCTTATTGATATGC-3′), translation elongation factor 1-alpha (TEF-1α) using primers EF1/EF2 (EF1: 5′-ATGGGTAAGGAGGACAAGAC-3′, EF2: 5′-GGAAGTACCAGTGATCATGTT-3′), and RNA polymerase II beta subunit (RPB2) using primers 5F2/7cR (5F2: 5′-GGGGWGAYCAGAAGAAGGC-3′, 7cR: 5′-CCCATRGCTTGYTTRCCCAT-3′) (Chen et al., 2020; Husna et al., 2020; Long et al., 2021). The amplicons were sequenced by Sangon Corporation (Shanghai, China) and deposited in the National Center for Biotechnology Information (NCBI, https://www.ncbi.nlm.nih.gov/) database under the accession numbers PP738014.1, PP780439.1, and PP780438.1, respectively. The DNA sequences of the isolates were analyzed for sequence similarity using the Basic Local Alignment Search Tool (BLAST) program against the NCBI database. A phylogenetic tree based on the ITS, TEF-1α, and RPB2 sequences was constructed using the neighbor-joining method implemented in MEGA version 11.0 software.

### 2.4 Inhibition activity of carvacrol against the specific pathogen

#### 2.4.1 *In vitro* antifungal activity test

The inhibition activities of carvacrol against the specific pathogen at different concentrations (25, 50, 75, 100, 125, and 150 μg/L) were determined using the mycelium growth rate method (Li et al., 2024). Different quality of carvacrol were dissolved in 1 mL of dimethylsulfoxide (DMSO) and then mixed with 9 mL of 0.1% Tween 20 solution and 90 mL of PDA medium. Subsequently, the mixture was poured into 3 dishes and allowed to cool to room temperature for the preparation of PDA plates. Mycelia dishes of the pathogen with an approximate diameter of 0.4 cm were excised from the culture medium and aseptically transferred to the center of each PDA plate using a sterile inoculation needle. The inoculated PDA plates were incubated at 28 °C for a period of 4 days. DMSO was used as a negative control, while prochloraz was used as a positive control. The inhibition rates of carvacrol and prochloraz at different concentrations were calculated using the established method (Chattapadhyay and Dureja, 2006). The median effective concentration (EC_50_) values were also calculated via the GraphPad Prism Software (San Diego, USA). The experiment was conducted in triplicate.

#### 2.4.2 Effect of carvacrol on the hyphae morphology

The specific pathogen was cultured on a PDA plate supplemented with a median effective concentration (EC_50_) concentration of carvacrol, while the pathogen treated with DMSO served as the negative control. The experiment was conducted in triplicate. Following a 24 h incubation at 28 °C, the hyphae samples were fixed in 2.5% glutaraldehyde at room temperature for 24 h, then washed three times with 0.1 M phosphate buffer for 15 min each, followed by a 1 h fixation in 1% OsO_4_ solution. Then the specimens were dehydrated in a gradient ethanol series (20, 50, 80, and 100%, respectively, 5 min for each alcohol dilution). After drying at critical point and gold coating, scanning electron microscope (SEM; Hitachi Ltd., Tokyo, Japan) observations on the hyphae morphology of the specific pathogen were conducted (Suzuki et al., 2017).

### 2.5 Transcriptome and proteomics analysis

The specific pathogen was cultured on a PDA plate supplemented with an EC_50_ concentration of carvacrol (designated as FX), while the pathogen treated with DMSO served as the negative control (designated as FC). Following a 72 h incubation at 28 °C, the hyphae of FX and FC samples were collected for transcriptome and proteomics analysis.

Transcriptome sequencing of the hyphae was conducted by Hangzhou Lianchuan Biological Co., Ltd., using the Illumina HiSeq™ 2000 platform (Illumina Inc., San Diego, CA, USA). The raw sequence data have been deposited in the National Center for Biotechnology Information (NCBI) database under the accession number PRJNA1195909. To ensure high-quality reads, cutadapt software (v1.9.3) was employed to filter out low-quality reads and hisat2 software (v2.0.4) was utilized to align high quality clean reads against the reference genome (Kechin et al., 2017). Differentially expressed genes (DEGs) were identified using an R language package, with a significance threshold of *p* < 0.05 and a log_2_FC > 1 (Sui et al., 2023).

Proteomics sequencing of the hyphae were analyzed using a liquid chromatography tandem–mass spectrometry (LC–MS/MS) system (5,600 Triple TOFMS) coupled with a Nano-Liquid Chromatograph (Eksigent, Dublin, CA, USA) (Teng et al., 2021). The raw data were deposit to ProteomeXchange Consortium (http://proteomecentral.proteomexchange.org) with the accession number of PXD057043. The raw data were quantified by the MaxQuant software (version 1.5.8.3) (Cox et al., 2011). Differentially expressed proteins (DEPs; expression level > 2.0-fold, *p* < 0.01) were identified from the Uniprot database (http://www.uniprot.org/) (Gao et al., 2017).

Gene Ontology (GO) annotations, encompassing biological processes (BP), cellular components (CC), and molecular functions (MF), as well as Kyoto Encyclopedia of Genes and Genomes (KEGG) pathway enrichments for the DEGs and DEPs were performed at http://www.geneontology.org/ and https://www.kegg.jp/kegg/pathway.html, respectively (Yu et al., 2018; Zhang et al., 2023).

## 3 Results

### 3.1 Preservative effects of carvacrol on the postharvest rot in garlic scapes

**Figure 1:**
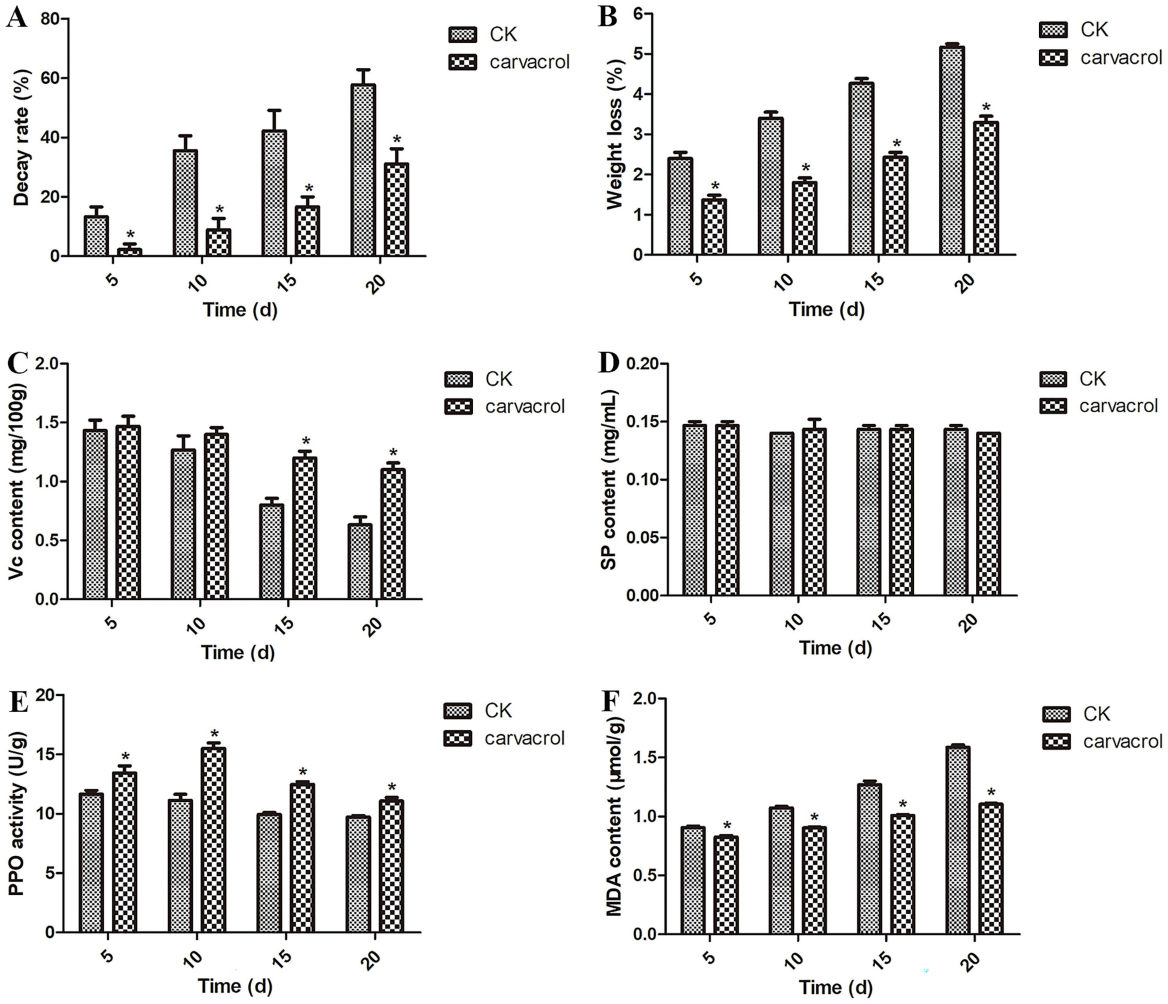
Effect of carvacrol on the decay rate **(A)**, weight loss **(B)**, Vc content **(C)**, SP content **(D)**, PPO activity **(E)**, and MDA content **(F)** at 5, 10, 15 and 20 days post-treatment, respectively. U, active unit. Vertical bars represent the standard errors of the means. Asterisk (*) means significantly different among deferent treatment group at a significance level of *p* < 0.05.

Figure 1A shows that, at 20 days post-treatment, the decay rate in the control group reached 57.78%, whereas it was only approximately 31.11% in the carvacrol-treated group. These results suggest that carvacrol effectively inhibits postharvest decay, thereby extending the shelf life of garlic scapes. Figure 1B shows that during storage, weight loss increased in all groups; however, the carvacrol-treated group exhibited significantly lower weight loss compared to the control (CK) group. Figure 1C also demonstrates that carvacrol treatment significantly delayed the decrease in Vc content in garlic scapes relative to the CK group. Figure 1D shows that carvacrol treatment had no significant effect on SP content. Figure 1E indicates that carvacrol treatment significantly enhanced PPO activity, with the highest level (15.50 U/mg) observed at 10 days post-treatment. Additionally, Figure 1F shows that carvacrol treatment significantly inhibited the increase in MDA content, thus delaying spoilage of postharvest garlic scapes.

### 3.2 Preservative effects of carvacrol on the postharvest rot in garlic scapes

**Figure 2:**
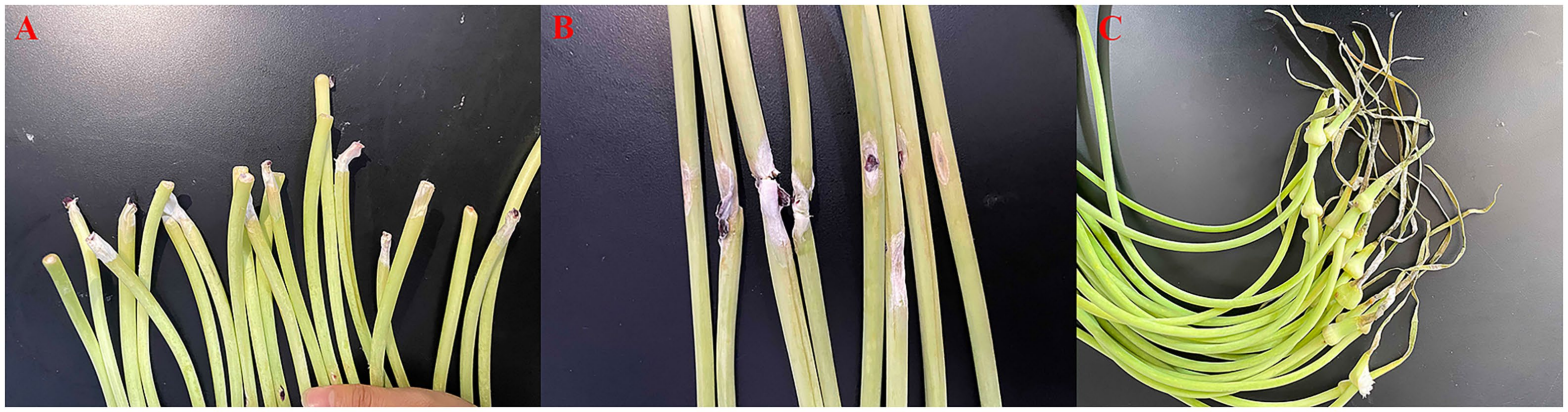
Symptoms of the basal **(A)**, stem **(B)**, and apical **(C)** regions of garlic sprouts after inoculation with *F* strain.

A total of eight fungi [PQ (*Penicillium* spp.), LS (*Trichoderma* spp.), F (*Fusarium* spp.), HJ (*Fusarium* spp.), BS (*Irpex* spp.), BX (*Bjerkandera* spp.), HB (*Mucor* spp.), and HQ (*Aspergillus* spp.)] with different morphology were isolated from the infected base, stem, and apical tissues of symptomatic garlic scapes. The pathogenicity tests of the isolated eight fungi were conducted by inoculating a conidial suspension (1.0 × 10^6^ conidia/L) onto the basal, stem, and apical regions of 20 fresh garlic scapes (“Chaohua” cultivar). After an incubation period of 7 days in an incubator set at 28 °C with 95% relative humidity, as shown in Figure 2, rot symptoms infected by F strain (infection rate 60%) resembling those observed in the collected samples were observed in the base, stem, and apical of garlic scapes.

**Figure 3:**
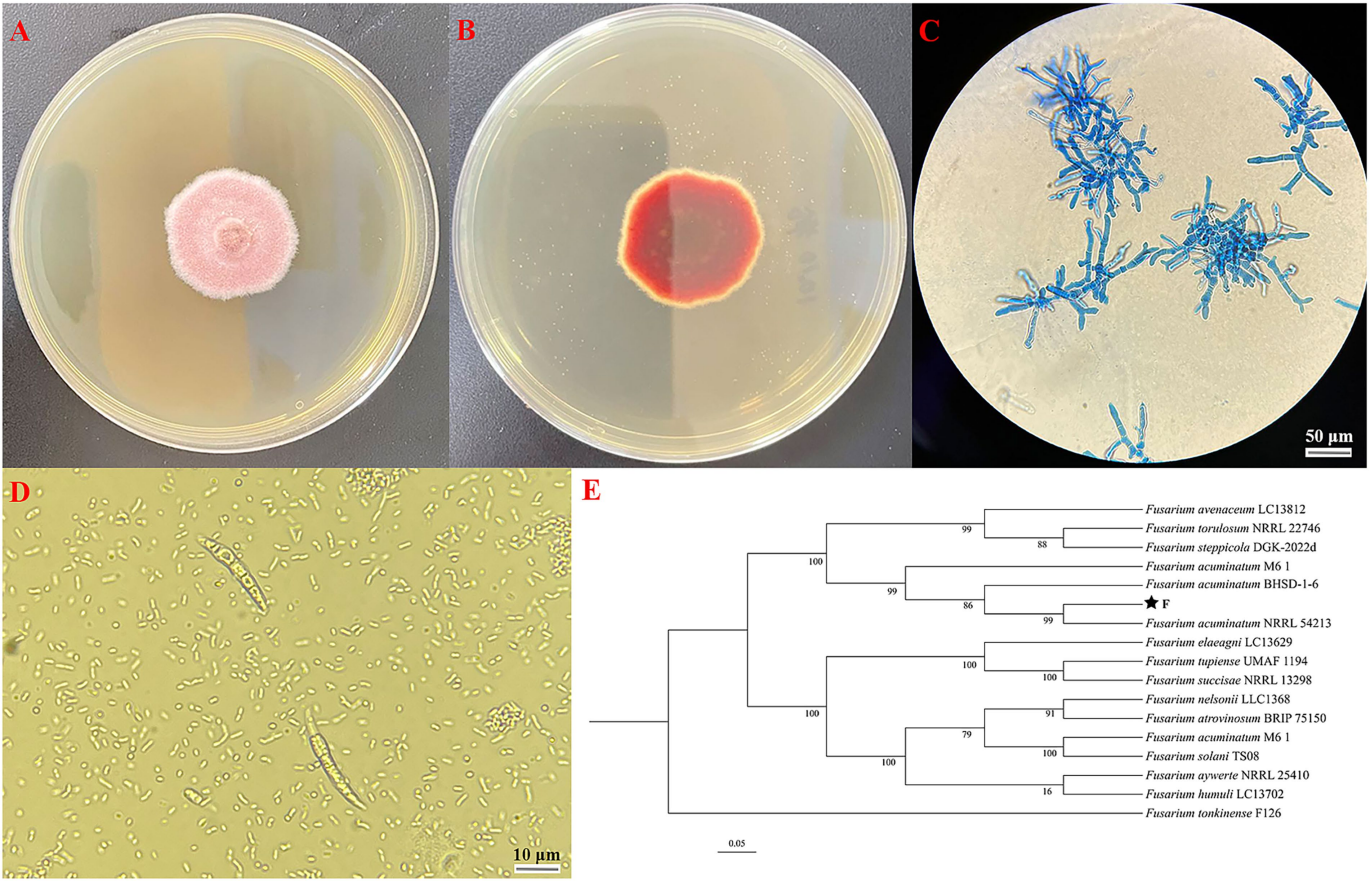
**(A)** Observe surface of *F* strain incubation on front side of PDA plate; **(B)** observe surface of F strain incubation on back side of PDA plate; **(C)** morphology of hyphae of *F* strain; **(D)** morphology of conidia of *F* strain; **(E)** phylogenetic tree analysis based on the PCR sequence of F strain.

The F strain re-isolated from the symptomatic garlic scapes based on Koch’s postulates was subsequently selected for further characterization through morphological and molecular biological characterization. The mycelium exhibits a flocculent appearance, with the front displaying a light pink hue and the back ranging from light pink to reddish purple, occasionally exhibiting concentric ring growth patterns (Figures 3A,B). Additionally, the optical microscope revealed that the mycelium exhibits branching and septation; the conidial stalk displays a branching structure resembling a slender bottle-shaped stem, which bears large crescent-shaped conidia on 1–5 compartments, with 1–3 being the most prevalent (Figures 3C,D). Utilizing MEGA version 11.0 software with the neighbor-joining method, a phylogenetic tree was generated which revealed a complete match of 99% homology between *F* strain and *Fusarium acuminatum* NRRL54213 (Figure 3E). Consequently, through the integration of morphological characterization and molecular biological identification, the *F* strain was definitively identified as *Fusarium acuminatum*.

### 3.3 *In vitro* antifungal activity

As illustrated in Table 1, the inhibition rates of carvacrol against *Fusarium acuminatum* exhibited a significant dose-dependent increase, reaching 39.98, 52.48, 82.06, 88.80, 100.00, and 100.00% at concentrations of 25, 50, 75, 100, 125, and 150 μg/L, respectively. Meanwhile, the EC_50_ value for carvacrol against *Fusarium acuminatum* was determined to be 36.17 μg/L, which was even better than that of prochloraz, suggesting that carvacrol exhibits potent *in vitro* antifungal activity against this pathogen.

**Table 1:** The *in vitro* antifungal activity of carvacrol against *Fusarium acuminatum*.

Treatments	Concentration (μL/L)	Inhibition rate (%)	EC_50_ (μg/L)
Carvacrol	25	39.98 ± 2.35	36.17 ± 1.65
	50	52.48 ± 1.63	
	75	82.06 ± 2.65	
	100	88.80 ± 2.15	
	125	100.00	
	150	100.00	
Prochloraz	25	36.15 ± 1.56	39.37 ± 0.25
	50	19.56 ± 2.21	
	75	76.54 ± 1.07	
	100	86.62 ± 1.19	
	125	98.26 ± 2.05	
	150	100.00	

### 3.4 Effect on the hyphae morphology

**Figure 4:**
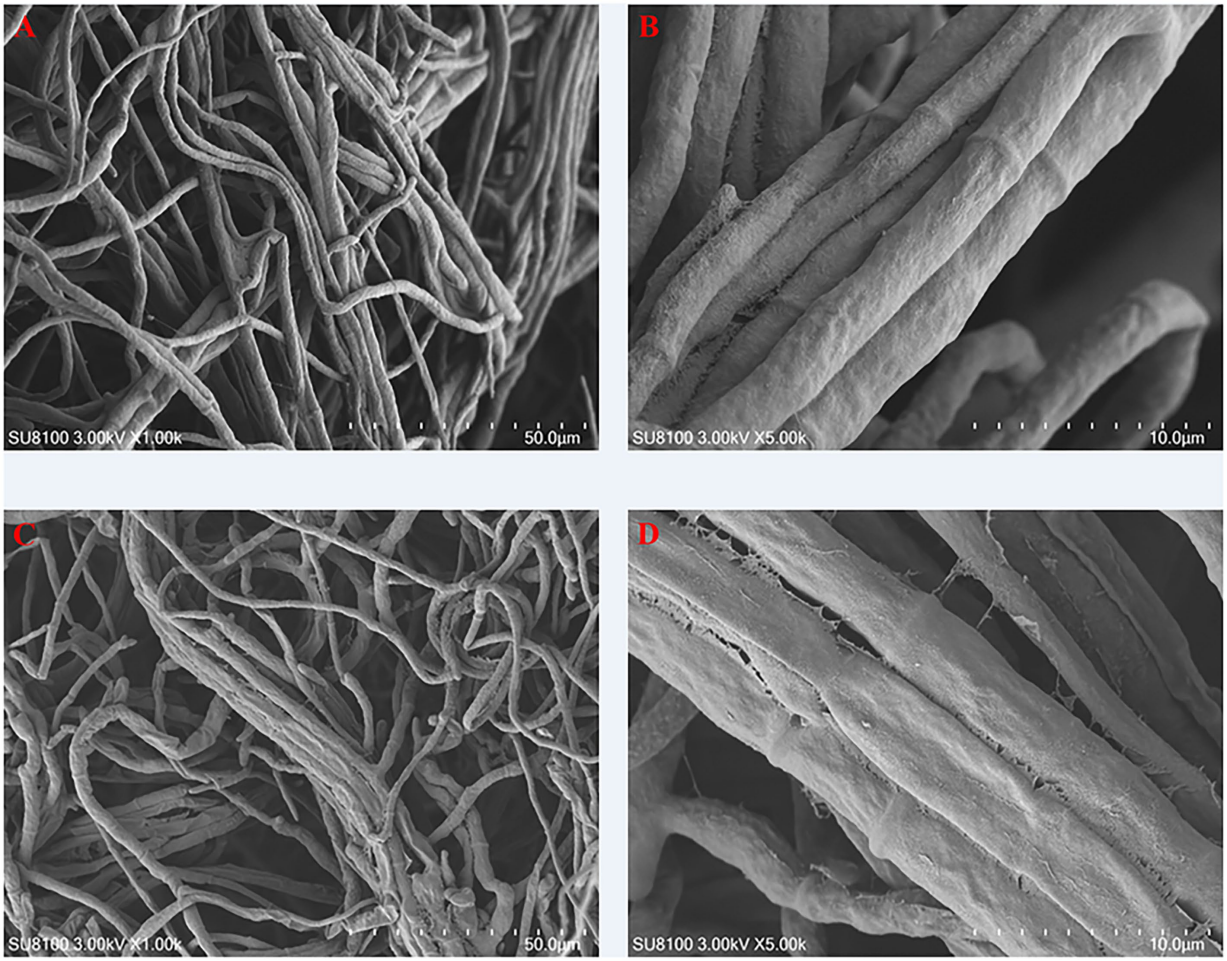
SEM observations on the hyphae morphology of *Fusarium acuminatum* treated by DMSO **(A,B)** and carvacrol **(C,D)**.

SEM was employed to investigate the impact of carvacrol on the microstructure of *Fusarium acuminatum*. The findings, as illustrated in Figures 4A,B, revealed that the hyphae surface in the control group exhibited regular fullness and maintained a normal physiological structure. In contrast, as illustrated in Figures 4C,D, the hyphae in the treatment group displayed irregular contractions, pronounced folds, depressions, and shriveled areas on the hyphal surface, with some hyphal fragments breaking off. These observations indicate that carvacrol treatment induced irreversible alterations in the morphology and structure of the hyphae, leading to deformation and rupture, thus demonstrating a certain inhibitory effect on *Fusarium acuminatum*.

### 3.5 Quality check of transcriptome sequencing data

Table 2 shows that, after cleaning and quality checking, 58.51, 54.48, 55.71 and 58.48, 56.16, 51.90 Mb clean reads, with Q30 bases (base quality >30) contents ranging from 94.55 to 95.17% and GC contents ranging from 53.61 to 53.94%, were generated from the cDNA libraries of FX and FC samples, respectively. In general, the sequencing results are of good quality and the data can be used for subsequent bioinformatics analysis.

**Table 2:** Overview of transcriptome sequencing date.

Samples	Raw reads (Mb)	Clean reads (Mb)	GC content (%)	Clean reads ≥Q30 (%)
FX-1	58.12	58.51	53.94	95.15
FX-2	56.85	54.48	53.74	94.60
FX-3	60.48	55.71	53.61	94.77
FC-1	60.71	58.48	53.82	94.55
FC-2	58.28	56.16	53.91	95.17
FC-3	54.19	51.90	53.83	95.02

### 3.6 DEGs identification

**Figure 5:**
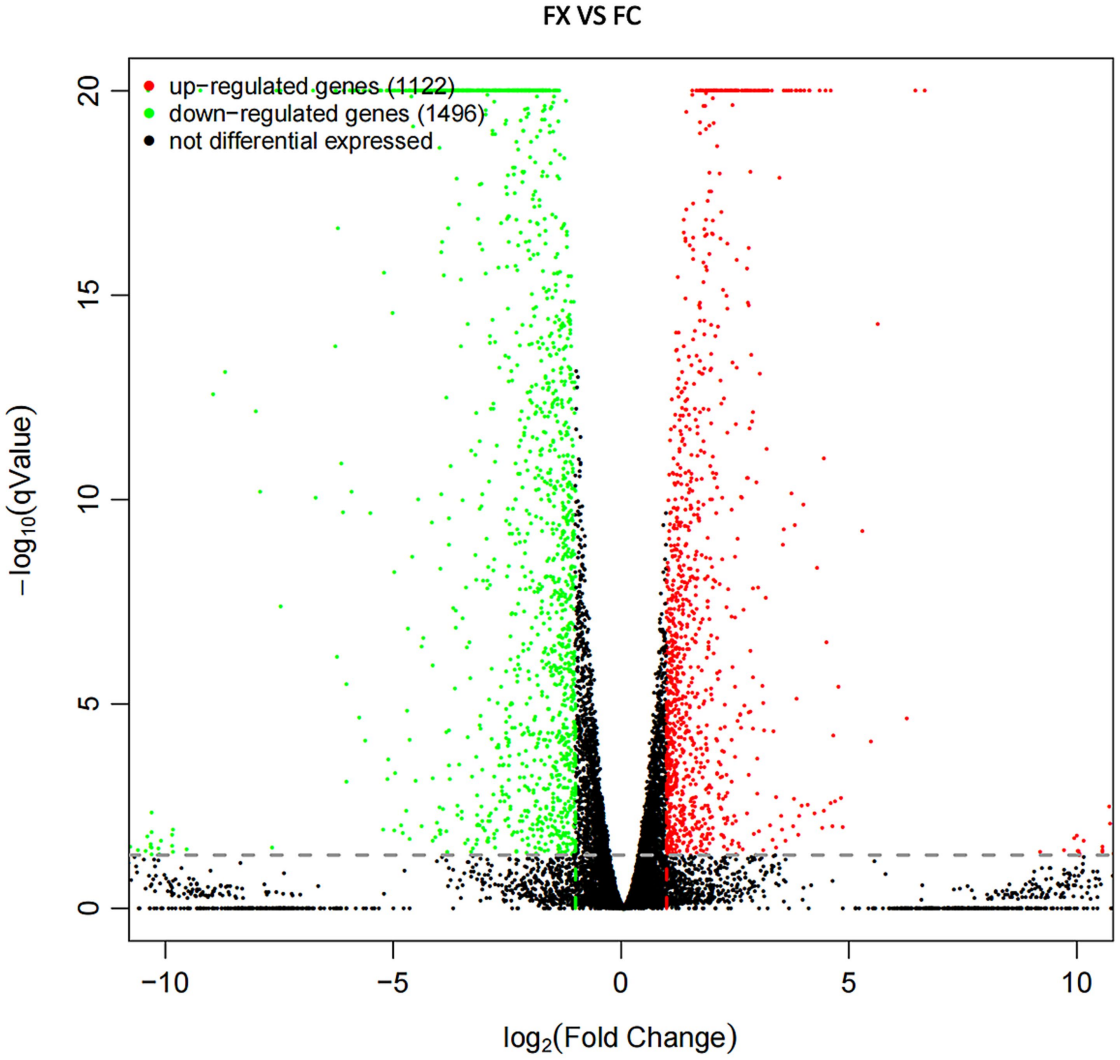
Volcano plot diagram of DEGs of FX vs. FC. The red points are significant up-regulated genes, the green points are significant down-regulated genes, while the black genes are not differential expressed genes.

Compared sample FX with FC, a total of 2,618 DEGs (including 1,122 up-regulated and 1,496 down-regulated genes) were detected (Figure 5 and Supplementary Table S1), of which the up- and down-regulated genes were 1857 and 2,114, respectively.

### 3.7 Bioinformatics analysis of DEGs

**Figure 6:**
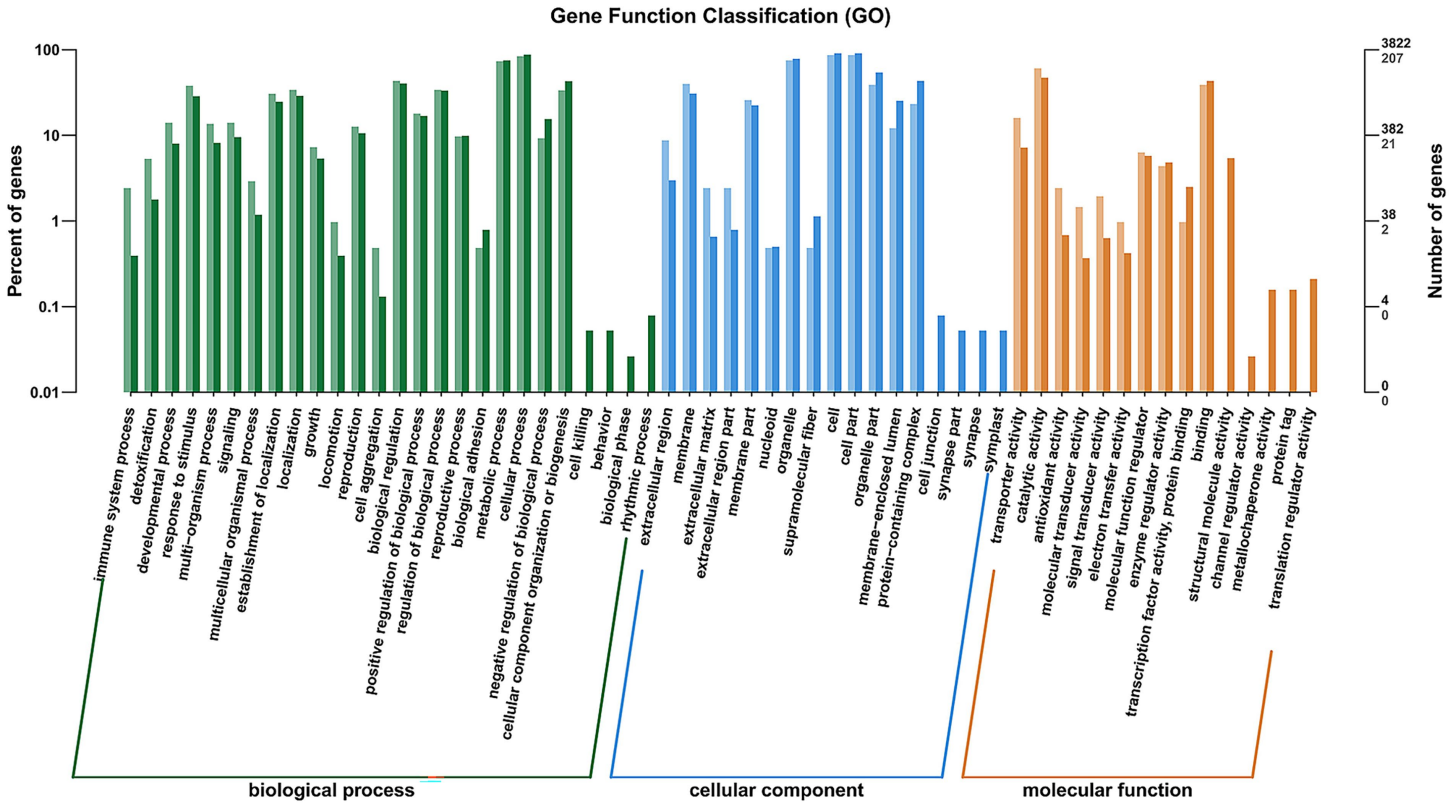
Go (*p* value <0.05 and the highest enrichment score calculated as the negative logarithm of the corresponding *p* value) term enrichment analysis of DEGs of FX vs. FC. *X*-axis represents different functional groups (also named as different GO terms), while *Y*-axis indicates the percentage that each functional group gene and accounts for the total genes, respectively.

To further functional characterization of the DEGs of FX vs. FC, GO analysis was classified and annotated into 3,167 known GO terms, comprising 70.25% (2,225 GO terms) in BP, 10.58% (335 GO terms) in CC, and 19.17% (607 GO terms) in MF (Supplementary Table S2). Go term enrichment analysis of FX vs. FC (Figure 6) demonstrated that the main BP involved immune system process, detoxification, developmental process, response to stimulus, multi-organism process, signaling, multicellular organismal process, establishment of localization, localization, growth, locomotion, reproduction, cell aggregation, biological regulation, positive regulation of biological process, regulation of biological process, reproductive process, biological adhesion, metabolic process, cellular process, negative regulation of biological process, cellular component organization or biogenesis, cell killing, behavior, biological phase, and rhythmic process. The main CC involved extracellular region, membrane, extracellular matrix, extracellular region part, membrane part, nucleoid, organelle, supramolecular fiber, cell, cell part, organelle part, membrane-enclosed lumen, protein-containing complex, cell junction, synapse part, synapse, and symplast. The main MF involved transporter activity, catalytic activity, antioxidant activity, molecular transducer activity, signal transducer activity, electron transfer activity, molecular function regulator, enzyme regulator activity, transcription factor activity, protein binding, binding, structural molecule activity, channel regulator activity, metallochaperone activity, protein tag, and translation regulator activity.

**Figure 7:**
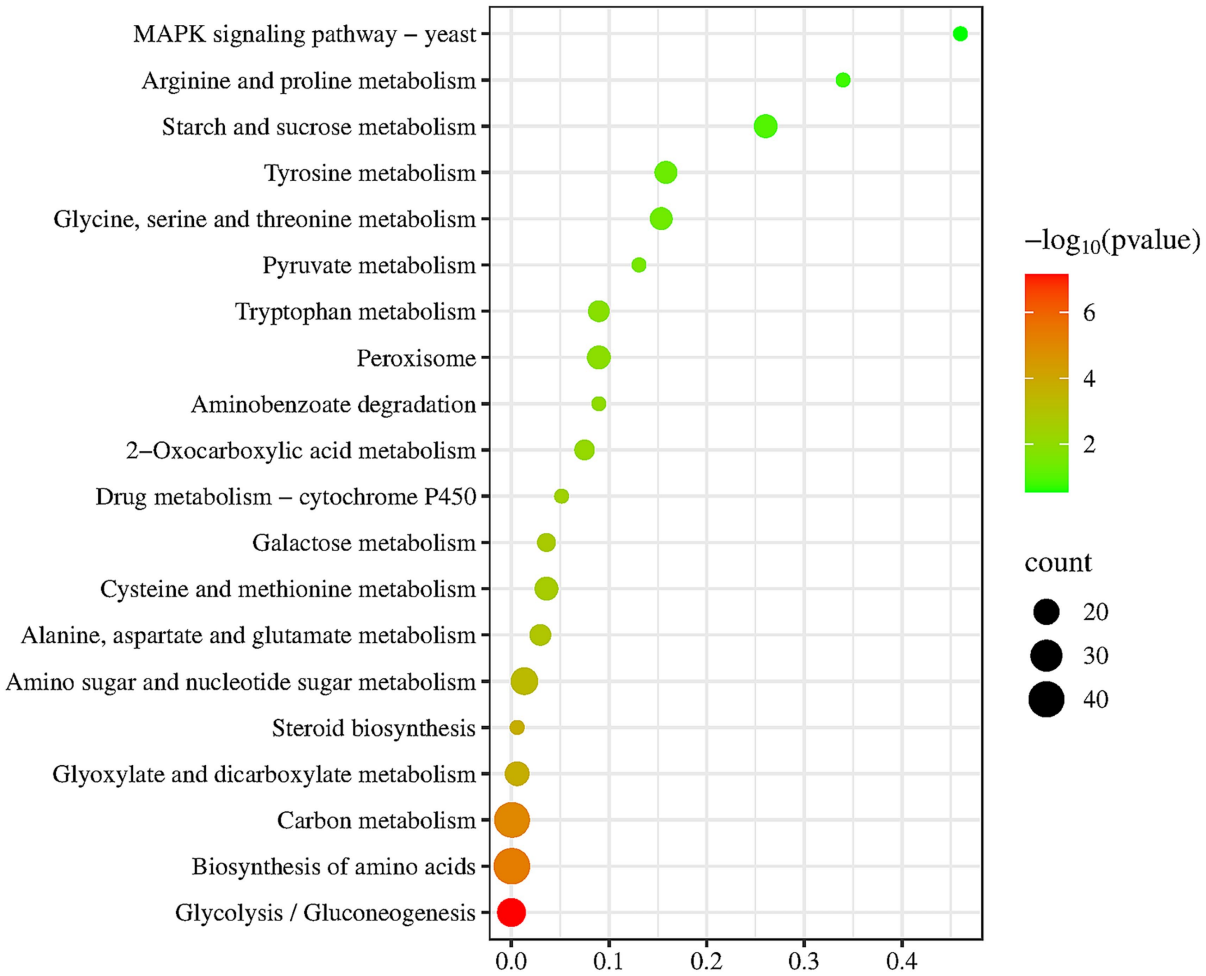
Top twenty KEGG pathways enrichment of DEGs of FX vs. FC. The depth of color reflects the level of significance, as indicated by the corresponding color legend on the side. The size of the bubbles represents the scale of enrichment, with larger bubbles indicating a greater number of DEGs enriched in the given pathway.

To further functional characterization of the DEGs of FX vs. FC, pathway analysis based on the KEGG database was classified and annotated into 235 known KEGG pathways (Supplementary Table S3). KEGG pathways analysis of FX vs. FC (Figure 7) revealed that DEGs were mainly annotated into MAPK signaling pathway, arginine and proline metabolism, carbon metabolism, biosynthesis of amino acids, and steroid biosynthesis.

### 3.8 DEPs identification

**Figure 8:**
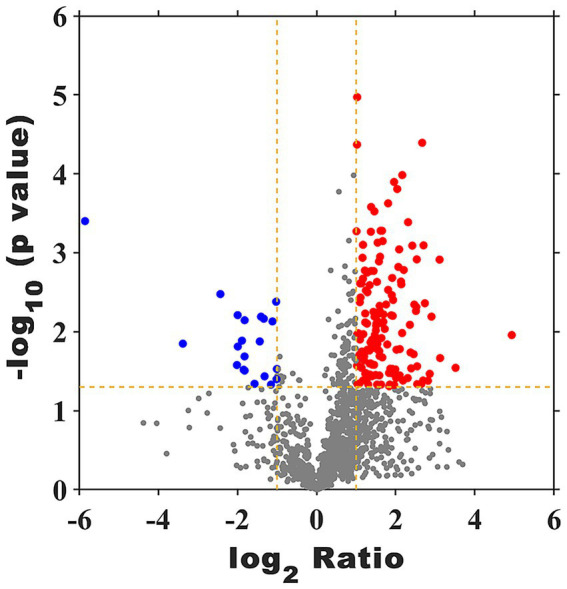
Volcano plot of DEPs of FX vs. FC. The red points are significant up-regulated genes, the blue points are significant down-regulated genes, while the black genes are not differential expressed genes.

As shown in Figure 8 and Supplementary Table S4, a total of 1862 proteins were identified and the up- and down-regulated proteins in FX vs. FC were 147 and 21, respectively.

### 3.9 Bioinformatics analysis of DEPs

**Figure 9:**
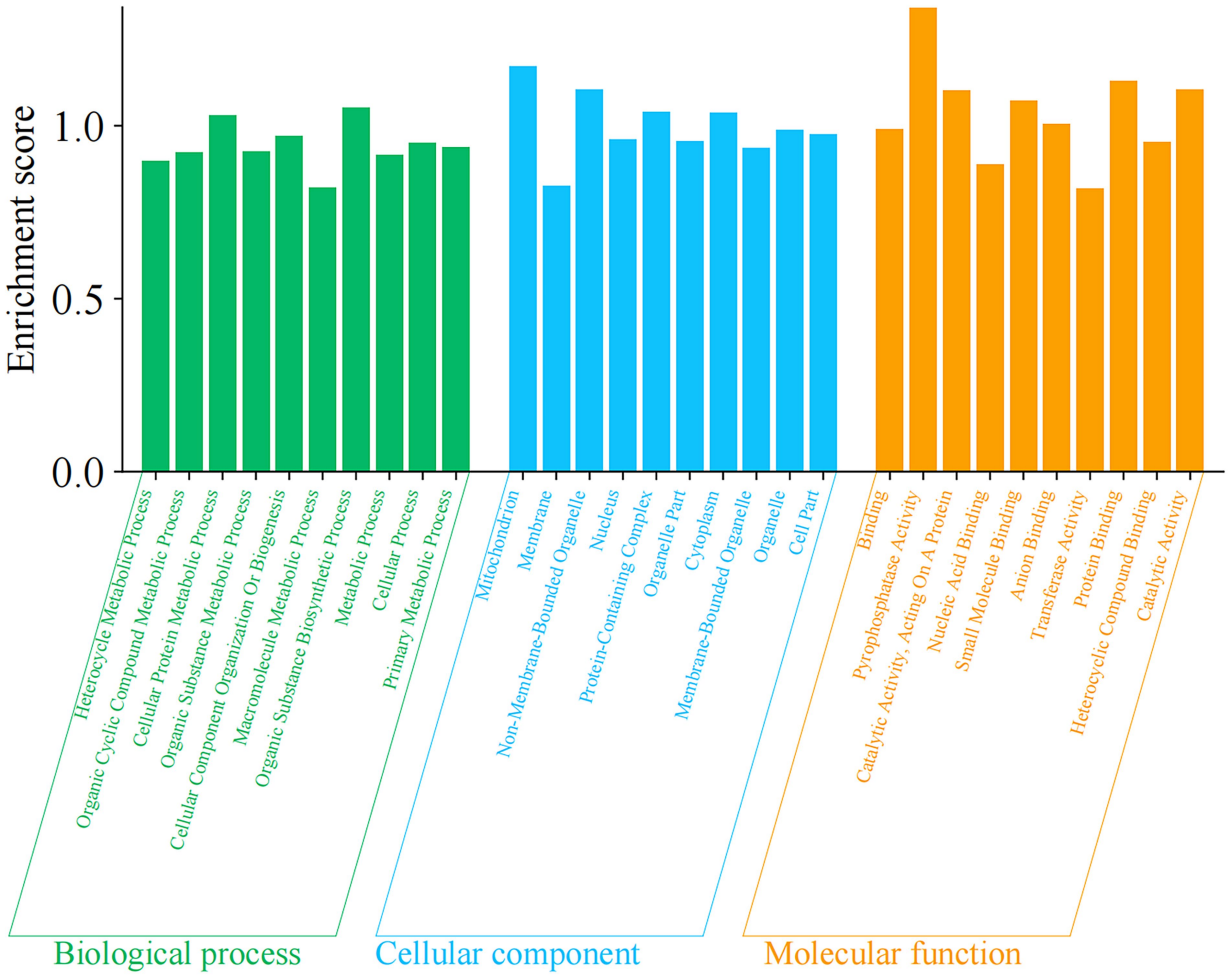
Go (*p* value <0.05 and the highest enrichment score calculated as the negative logarithm of the corresponding *p* value) term enrichment analysis of DEPs of FX vs. FC. *X*-axis represents different functional groups (also named as different GO terms), while *Y*-axis indicates enrichment score.

The GO analysis of the DEPs was annotated into 10,212 known GO terms, comprising 64.43% (6,579 GO terms) in BP, 12.11% (1,237 GO terms) in CC, and 23.46% (2,396 GO terms) in MF (Supplementary Table S5). Go term enrichment analysis of FX vs. FC (Figure 9) demonstrated that the main BP involved heterocycle metabolic process, organic cyclic compound metabolic process, cellular protein metabolic process, organic substance metabolic process, cellular component organization or biogenesis, macromolecule metabolic process, organic substance biosynthetic process, metabolic process, cellular process, and primary metabolic process. The main CC involved mitochondrion, membrane, non-membrane-bounded organelle, nucleus, protein-containing complex, organelle part, cytoplasm, membrane-bounded organelle, organelle, and cell part. The main MF involved pyrophosphatase activity, binding, catalytic activity, acting on A protein, nucleic acid binding, small molecule binding, anion binding, transferase activity, protein binding, heterocyclic compound binding, and catalytic activity.

**Figure 10:**
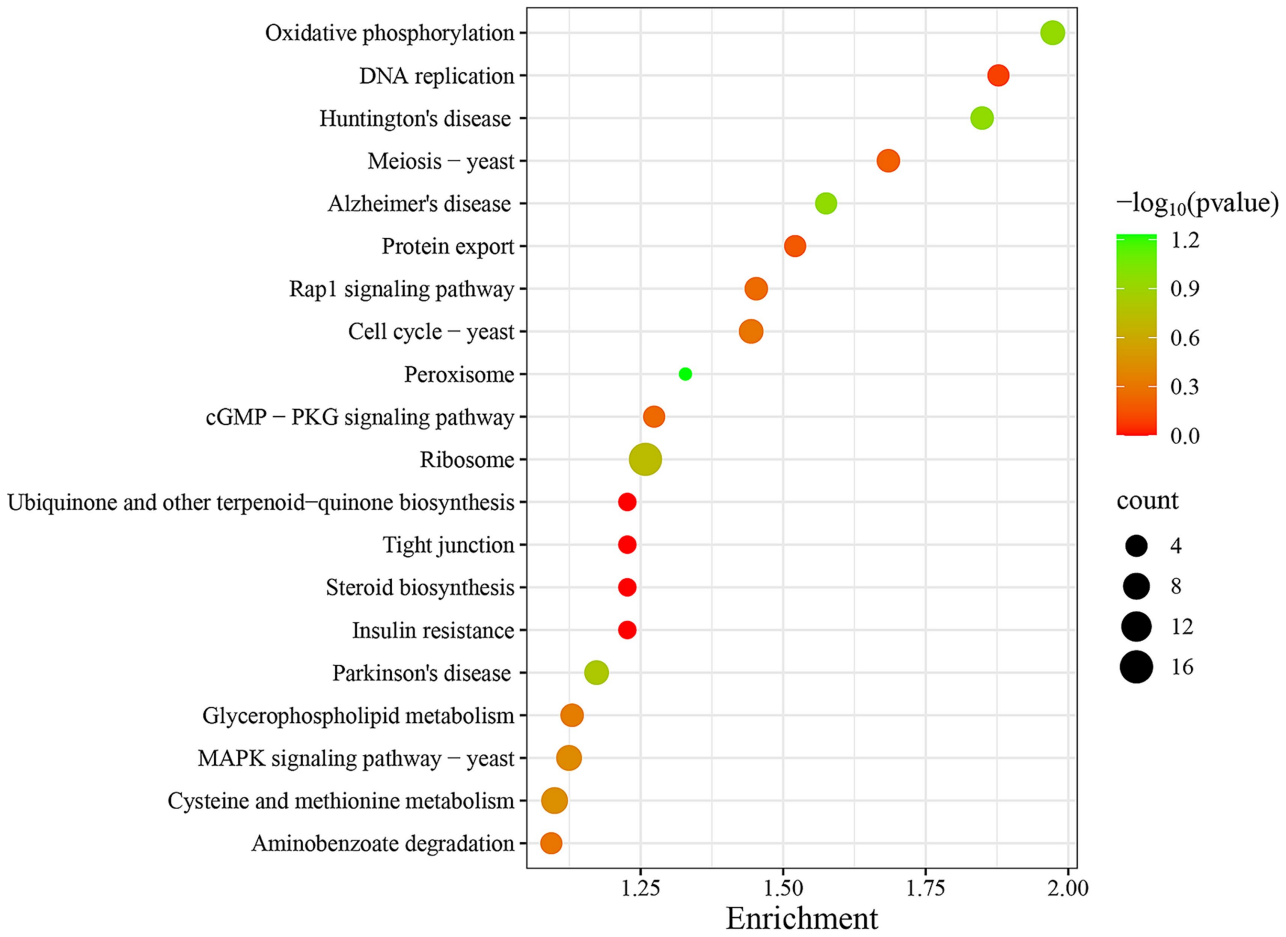
Top twenty KEGG pathways enrichment of DEPs of FX vs. FC. The depth of color reflects the level of significance, as indicated by the corresponding color legend on the side. The size of the bubbles represents the scale of enrichment, with larger bubbles indicating a greater number of DEPs enriched in the given pathway.

To further functional characterization of the DEPs of FX vs. FC, pathway analysis based on the KEGG database was classified and annotated into 1865 known KEGG pathways (Supplementary Table S6). KEGG pathways analysis of FX vs. FC (Figure 10) revealed that DEPs were mainly annotated into steroid biosynthesis, oxidative phosphorylation, ribosome, DNA replication, and MAPK signaling pathway.

## 4 Discussion

Carvacrol is a monoterpenic phenol produced by an abundant number of aromatic plants, including thyme and oregano (Veldhuizen et al., 2006; Mączka et al., 2023). Presently, carvacrol is used in low concentrations as a food flavoring ingredient and preservative to enhance the shelf life and safety of perishable foods, such as fermented pepper, fruit juice, and fresh-cut fruits (Yang et al., 2024). The weight loss in postharvest fruits and vegetables occurs during storage primarily due to respiration, moisture loss, and oxidation processes. Additionally, decay and mold can cause water loss, which contributes to the overall weight reduction (Owolabi et al., 2021; Singh et al., 2021). Vc, one of the most abundant water-soluble antioxidants in plants and animals, is of vital importance to human health and plays an important role in the defense of diseases related to collagen synthesis and protection against oxidative stress (Padayatty et al., 2003). As plant-based foods constitute the principal source of Vc in the human diet, the possibility of increasing the Vc content of plants to improve their nutritional value has also received considerable attention in recent years (Paciolla et al., 2019). Any changes in the Vc content of plant cells can result in a diverse range of effects on growth, development and stress tolerance, as Vc is involved in redox signalling, cell cycle regulation, enzyme functioning and the expression of defence and stress-related genes (Jiang et al., 2022). Hence, adequate intake of Vc from foods is necessary for normal physiological functioning, and fruits and vegetables are the richest natural sources of Vc in the human diet. PPO-catalyzed browning reactions, which occur in a wide range of plant-derived foods, significantly contribute to quality degradation and loss of nutritional value in the fruit and vegetable industry (Yourk and Marshall, 2007). A deeper understanding of the factors influencing PPO activity is crucial for effectively controlling and mitigating its adverse effects on plant-based products. MDA is a major byproduct of cellular membrane lipid peroxidation, which can induce cross-linking reactions in proteins, polysaccharides, nucleic acids, and other macromolecules. This biomarker effectively reflects the extent of potential damage to biological membranes (Mi et al., 2023). Herein, we observed that carvacrol treatment significantly delays the onset of rot symptoms in the basal, stem, and apical regions of garlic scapes compared to the control group. This suggests that carvacrol exerts a favorable postharvest preservative effect on garlic sprouts by delaying the decline in Vc content, enhancing PPO activity, and inhibiting the accumulation of MDA, thereby retarding the spoilage process.

In this study, we found that carvacrol exhibits potent *in vitro* inhibitory activity against *Fusarium acuminatum*, with an EC₅₀ value of 36.17 μg/L, which is even lower than that of prochloraz. These findings are supported by many previous studies. Zhang et al. (2019) demonstrated that carvacrol may serve as a promising alternative to conventional fungicides for controlling *Botrytis cinerea*-induced gray mold in horticultural products. Similarly, Šimović et al. (2014) reported that carvacrol exhibited significant inhibitory effects against foodborne pathogens such as *Aspergillus carbonarius* and *Penicillium roqueforti*, thereby enhancing the safety of fresh-cut watermelon. Meanwhile, in the present experiment, SEM observations indicate that carvacrol treatment induced irreversible alterations in the morphology and structure of the hyphae, leading to deformation and rupture, as reported by damaging cell membrane of *Botrytis cinerea* and *Rhizopus stolonifer* (Zhang et al., 2019; Jiang et al., 2015).

The mechanism of action of carvacrol against *Fusarium acuminatum* was then investigated utilizing the combined transcriptome and proteome analysis. The results showed that carvacrol mainly affected the steroid biosynthesis and MAPK signaling pathway cell signaling pathways in *Fusarium acuminatum*. In the steroid biosynthesis cell signaling pathway, ergosterol, a highly specific component of the fungal cell membrane, is synthesized (Beni et al., 2014). Ergosterol not only regulates membrane fluidity but is also essential for the formation and function of the plasma membrane, influencing the fluidity, permeability, and activity of cell membrane-associated proteins (Hu et al., 2017; Sun et al., 2020). A reduction in ergosterol synthesis can result in membrane dysfunction, thereby inhibiting fungal growth and reproduction. Under the influence of carvacrol, both lanosterol synthase (LSS) and sterol-4α-carboxylate 3-dehydrogenase (NSDHL) showed a significant downregulation during this regulatory process. The alterations in the expression levels of these enzymes can result in reduced ergosterol synthesis, thereby compromising the structural integrity and stability of cell membranes and inhibiting microbial growth and reproduction (Sayari et al., 2021). Meanwhile, the MAPK signaling pathway plays a critical role in cell proliferation and apoptosis, regulating various physiological processes including cell proliferation and apoptosis (Patergnani et al., 2020; Yue and López, 2020). Under the influence of carvacrol, the expression of guanylate binding protein (GBP) is downregulated, potentially impacting the cell cycle progression and resulting in a deceleration or cessation of cell proliferation, thus affecting the development of *Fusarium oxysporum* (Guo et al., 2020). Similar results reported by Chavan and Tupe (2014) demonstrated that carvacrol exerted its antimicrobial action against wine spoilage yeasts through membrane damage, leakage of cytoplasmic content and ergosterol depletion.

This study only focused on the preliminary mechanism of action of carvacrol against *Fusarium acuminatum* based on the integrated transcriptomic and proteomic analyses. The observed downregulation of key enzymes and signaling components suggests a potential link to the antifungal activity of carvacrol will be conducted in our future research.

## 5 Conclusion

In this study, we found that carvacrol can significantly delay the onset of postharvest rot symptoms of garlic scapes by delaying the decline in Vc content, enhancing PPO activity, and inhibiting the accumulation of MDA. Meanwhile, a specific pathogen causing postharvest rot of garlic scapes, identified as *Fusarium acuminatum*, was isolated from symptomatic garlic scapes. Our findings revealed that carvacrol demonstrated significant inhibitory activity against *Fusarium acuminatum*. SEM observations reveal that carvacrol treatment causes irreversible changes in the morphology and structure of hyphae, resulting in significant deformation and rupture. Moreover, the integrated transcriptome and proteome analysis revealed that carvacrol predominantly impacts the steroid biosynthesis and MAPK signaling pathway cell signaling pathways in *Fusarium acuminatum* to interference compromises the integrity and stability of the cell membrane, consequently suppressing the growth and proliferation of *Fusarium acuminatum*.

## 6 Future prospects

The incorporation of carvacrol as a food preservative can effectively inhibit the growth and proliferation of microorganisms responsible for postharvest decay, thereby enhancing preservation efficacy and extending the shelf life of fruits and vegetables in the postharvest food industry.

## Data Availability

The datasets presented in this study can be found in online repositories. The names of the repository/repositories and accession number(s) can be found in the article/Supplementary material.

## Supplementary material

SUPPLEMENTARY TABLE S1 List of DEGs of FX vs. FC.

SUPPLEMENTARY TABLE S2 The classified and annotated GO terms of DEGs of FX vs. FC.

SUPPLEMENTARY TABLE S3 KEGG pathways enrichment of DEGs of FX vs. FC.

SUPPLEMENTARY TABLE S4 List of DEPs of FX vs. FC.

SUPPLEMENTARY TABLE S5 The classified and annotated GO terms of DEPs of FX vs. FC.

SUPPLEMENTARY TABLE S6 KEGG pathways enrichment of DEPs of FX vs. FC.
